# Songbirds and humans apply different strategies in a sound sequence discrimination task

**DOI:** 10.3389/fpsyg.2013.00447

**Published:** 2013-07-17

**Authors:** Yoshimasa Seki, Kenta Suzuki, Ayumi M. Osawa, Kazuo Okanoya

**Affiliations:** ^1^ERATO Okanoya Emotional Information Project, Japan Science and Technology AgencyWako, Japan; ^2^Laboratory for Biolinguistics, Brain Science Institute, RIKENWako, Japan; ^3^Department of Life Sciences, Graduate School of Arts and Sciences, The University of TokyoTokyo, Japan

**Keywords:** rule-learning, sound sequences, operant conditioning, songbirds, Bengalese finches, artificial grammar

## Abstract

The abilities of animals and humans to extract rules from sound sequences have previously been compared using observation of spontaneous responses and conditioning techniques. However, the results were inconsistently interpreted across studies possibly due to methodological and/or species differences. Therefore, we examined the strategies for discrimination of sound sequences in Bengalese finches and humans using the same protocol. Birds were trained on a GO/NOGO task to discriminate between two categories of sound stimulus generated based on an “AAB” or “ABB” rule. The sound elements used were taken from a variety of male (M) and female (F) calls, such that the sequences could be represented as MMF and MFF. In test sessions, FFM and FMM sequences, which were never presented in the training sessions but conformed to the rule, were presented as probe stimuli. The results suggested two discriminative strategies were being applied: (1) memorizing sound patterns of either GO or NOGO stimuli and generating the appropriate responses for only those sounds; and (2) using the repeated element as a cue. There was no evidence that the birds successfully extracted the abstract rule (i.e., AAB and ABB); MMF-GO subjects did not produce a GO response for FFM and vice versa. Next we examined whether those strategies were also applicable for human participants on the same task. The results and questionnaires revealed that participants extracted the abstract rule, and most of them employed it to discriminate the sequences. This strategy was never observed in bird subjects, although some participants used strategies similar to the birds when responding to the probe stimuli. Our results showed that the human participants applied the abstract rule in the task even without instruction but Bengalese finches did not, thereby reconfirming that humans have to extract abstract rules from sound sequences that is distinct from non-human animals.

## Introduction

Abstract rule learning from sound sequences should be an essential factor in language acquisition of humans. Therefore, comparison of the ability to extract abstract rules from stimulus sequences between humans and non-human animals should be an interesting topic from a view point of human language evolution. To date, several researchers have reported such ability in animals and humans. Seven-month-old human infants can detect differences between sound sequences created from “AAB” (i.e., the first X is followed by the same X and then followed by different Y) and “ABB” type rules, indicating that they can extract “algebra-like rules” (Marcus et al., 1999). Rhesus monkeys also showed a competence for such rule extraction (Hauser and Glynn, 2009). In those human and monkey studies, spontaneous activities (i.e., visual attention of the subjects to the sound projecting loudspeaker) were taken as responses to the stimuli. Meanwhile, classical or instrumental conditioning techniques with food reinforcement have been generally applied in studies on this topic using rodents and songbirds. Murphy et al. (2008) reported that rats differentiated AAB, ABA, and BAA sequences, although the stimuli were simple combinations of only two types of stimuli: bright and dim light. A sophisticated study in zebra finches showed that birds learned to distinguish various sequences (including AAB and ABB); however, “algebra-like rules” were not used for discrimination (van Heijningen et al., 2009). Gentner et al. (2006) examined the ability to discriminate “syntactic” rules in European starlings, a songbird species that generates quite complex song sequences. They reported that the birds could discriminate between “grammatical” and “agrammatical” sequences in “center embedded sentences,” although alternative interpretations have been suggested for the results (van Heijningen et al., 2009; See also Gentner et al., 2010; ten Cate et al., 2010). Then, Abe and Watanabe (2011) reported that Bengalese finches (Lonchura striata var. domestica), which generate simpler songs than European starlings but more complex song sequences than zebra finches (Okanoya, 2004), showed spontaneous different vocal responses to playback of “ungrammatical” sounds after habituation to “grammatical” sounds when using a “center embedded sentences” rule, although alternative interpretations have also been suggested for those results (Beckers et al., 2012).

In studies of small animals like songbirds and rats, it should be difficult to detect visual attention of those subjects. Therefore, the discrimination tasks described above are appropriate methods for those studies. Meanwhile, to train human infants for discrimination tasks on an operant method using some reinforcements might be not appropriate, thus, the experimental methods used in previous infant studies should be adequate. However, researchers may have two questions regarding those studies for comparing the ability of rule extraction from sound sequences between human and animals: (1) how do the methods affect the results? (i.e., spontaneous response in a passive situation vs. conditioned behavior on active discrimination tasks); (2) how do species differences affect the results? (between primates and other species, or between zebra finches and other songbird species). To address some factors of those questions, we compared the discrimination strategies of Bengalese finches and humans presented with AAB and ABB sequences during an operant task. Hereafter, we use the term “rule-conforming” instead of grammatical and another term “non-rule-conforming” instead of “agrammatical” or “ungrammatical.”

## Materials and methods

### Experiment 1: bengalese finches

#### Subjects

Nine adult male birds (2–3 years old) were used; all birds were bred and maintained in our laboratory. Daily training and test sessions were done during day time (from forenoon to early afternoon). Food access of the birds was limited for 1–2 h around 4–6 pm but vitamin-enriched water and shell grit were available *ad libitum*. The light/dark cycle was 13/11 h. Room temperature and humidity were maintained at approximately 25°C and 60%, respectively. All experimental procedures and housing conditions were approved by the Animal Experiments Committee at RIKEN.

#### Apparatus

A test cage (W15.5 × D30.3 × H22.0 cm) was placed in a sound attenuation chamber (W89 × D70 × H74 cm, Music Cabin, Japan) that was illuminated by LEDs. The front panel of the cage had two response keys consisting of acrylic panels that could be accessed through 10 mm diameter holes; the left one served as the observation key, and the right one served as the report key. The keys were illuminated with a red and a green color when activated for pecking responses. A feeder was placed on the cage and delivered grains into a dish located 5 cm below the response keys. A small light illuminated the dish for 2 s when food was delivered. A loudspeaker was placed above the cage to deliver sound stimuli. A personal computer controlled the execution of the experiment.

#### Stimuli

**Figure 1:**
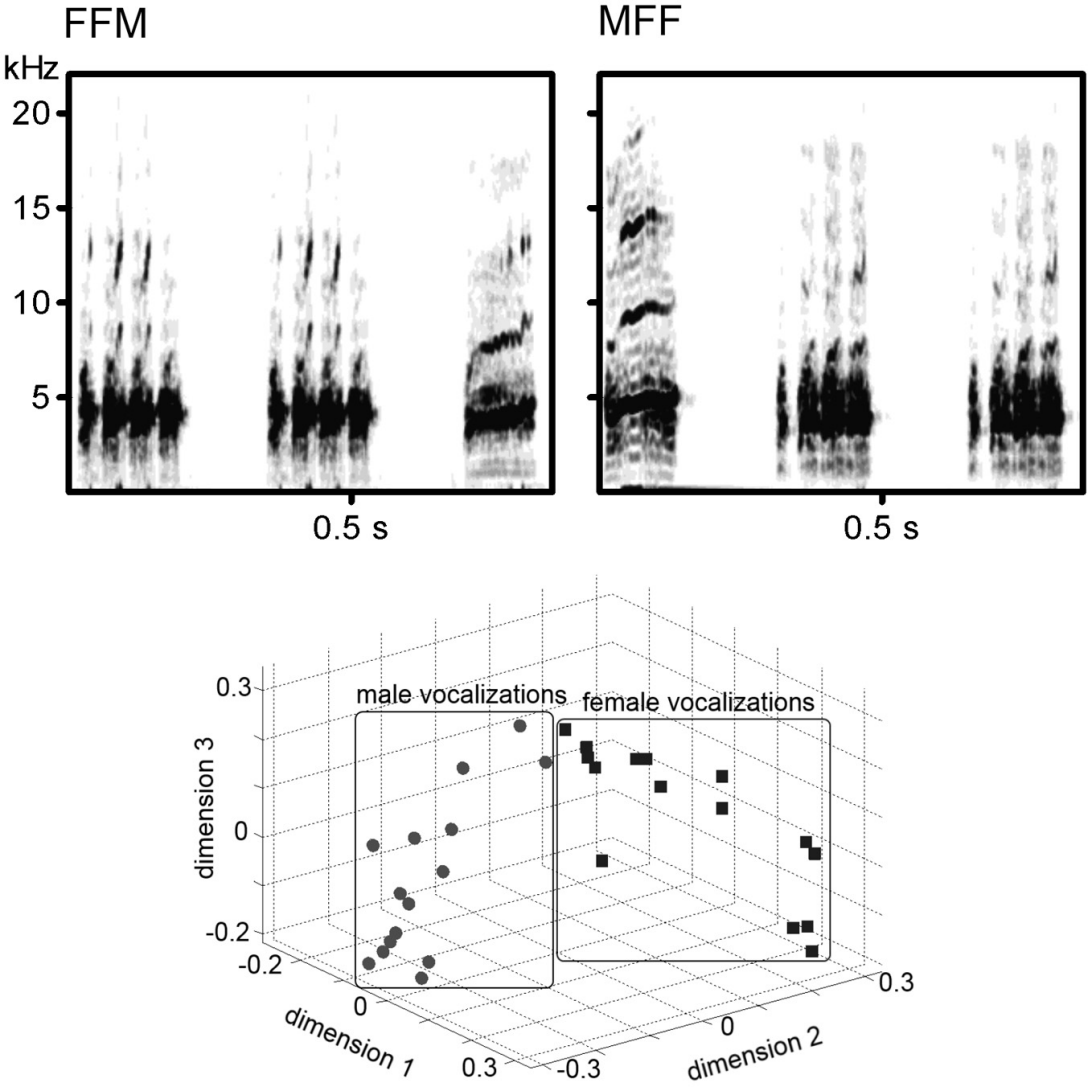
**(Upper panel) Examples of the spectrogram of the sound stimuli**. *Left*; Two calls from a female and a call from a male. *Right*; Two calls from another female and a call from another male. (**lower panel**). Scatter plots showing the similarity of the acoustic spectral patterns of male calls and female calls.

Distance calls of 15 adult male (M1–M15) and 15 adult female (F1–F15) Bengalese finches recorded in the sound attenuated box using Sound Analysis Pro (Tchernichovski et al., 2000) and digitized as.wav files (44.1 kHz, 16-bit) were stored as the stimulus database. The peak amplitude of each call (before making sound sequences) was adjusted to approximately 70 db SPL at the location of the bird's head. Those sounds were combined to create various stimuli: AAB (i.e., MMF or FFM) and ABB (MFF or FMM) sound sequences (how to choose the call sounds is described in the *Procedures* section). In each sequence, AA sequence was created from the same sound as well as each BB sequence (e.g., M1M1F5, F3M2M2). The inter-sound-interval was 100 ms in both A-B transitions and A-A/B-B repetitions. Examples of the sound spectrogram of the stimulus series were shown in upper panels of Figure 1. A previous study showed the difference between male and female calls was salient enough for the discrimination in Bengalese finches and such differences of the acoustic pattern were clearly appeared on the sound spectrograms (Okanoya and Kimura, 1993). In this study, we calculated the cross-correlation values of sound spectrograms among the 30 calls in a round-robin style (210 comparisons for within sex, 225 comparisons for inter-sexes). The value of within-sex was significantly larger than that of inter-sexes (*W* = 2931, *p* < 0.0001, Wilcoxon rank-sum test; lower panel of Figure 1 shows the result of a multi-dimensional scaling created from the cross-correlation values).

#### Procedures

First, birds were trained to peck the observation key using an auto-shaping method. Then, a GO/NOGO task was introduced requiring discrimination between AAB and ABB sequences. The observation key was activated to signal at the beginning of each trial. Following a key peck, a GO or NOGO stimulus was presented in semi-random order. The report key was activated after presentation of the sound stimulus. Pecking the report key within 2 s after a GO stimulus resulted in a food delivery (Hit). Otherwise, the bird did not obtain a reward for the trial (Miss). Meanwhile, pecking the report key within 2 s after a NOGO stimulus resulted in a punishment of black-out for 16 s (False Alarm; FA). Otherwise, the bird proceeded to the next trial without consequence (Correct Rejection; CR). Correction trials were applied for unsuccessful trials (FA or Miss) until the bird gave a correct response. The inter-trial interval was 4 s. Each training session concluded when 60 trials had been completed (30 each for GO and NOGO trial) or 40 min after starting the session.

The stimulus combination was counterbalanced across the birds (GO: MMF, *n* = 3; GO: FFM, *n* = 2; GO: MFF, *n* = 2; GO: FMM, *n* = 2). Five male and 5 female calls were randomly chosen from the database as training stimuli for each subject and combined to make 5 AAB and 5 ABB sequences. Neither GO nor NOGO trials were presented more than 4 times in a row. A correct response ((Hits + CRs)/(Hits + Misses + FAs + CRs) × 100) rate above 85% on two consecutive days was required for each subject to proceed to a test session. Although birds completed a total of 10 test sessions, their performance was confirmed using the same criterion between each test session.

Every bird had two types of test session; (1) transfer test; and (2) rule-generalization test. For the transfer test, novel sounds were selected from males and females that were not used for the training stimuli for each subject. Then five sound combinations were created in the same manner as the training stimuli and used as probe stimuli. For the rule-generalization test, five novel sounds from males and female calls were selected as for the transfer test; however, the positions of the M and F sound in the sequence were switched (i.e., if a bird was trained with GO-MMF and NOGO-MFF, then FFM (rule- conforming) and FMM (non-rule-conforming) were used as the probe stimuli). During a probe session, 12 probe trials were randomly interspersed among 48 normal training trials. Birds completed five test sessions, during which a unique probe stimulus was presented for both the first and the second tests. Thus, there were 60 responses for probe trials from each bird on each test. The responses for all probe trials were neither reinforced nor punished. During the task, if a subject did not respond to the observation key after 300 s, then a grain was delivered automatically to stimulate the subject.

### Experiment 2: humans

Eleven males and 5 females (18–29 years old) participated in this experiment. Five males and 3 females were assigned to human voice (HV) experiment and the 6 males and 2 females were assigned to bird vocalization (BV) experiment. Experimental procedures were approved by the ethical committee for experimental research involving human subjects at Graduate School of Arts and Sciences of The University of Tokyo.

#### Apparatus

A monitor and speakers of a laptop computer was used for stimulus presentation. The response key was either the space-key or the enter-key of the laptop. The sound level was adjusted to be comfortable to hear for each participant.

#### Stimuli

The stimuli of HV were vocal sounds of “*na*” from 8 males (M) and 8 females (F) (NTT-Tohoku University Speech Data Set for Word Intelligibility Test based on Word Familiarity (FW03) and “Spoken Language” and the DSR Projects Speech Corpus (PASL-DSR, Itahashi, 1991), Speech Resources Consortium, National Institute of Informatics, Tokyo, Japan). The training and probe stimuli were created as the same as Experiment 1. Same procedure to create the stimuli used in Experiment 1 was used for BV experiment.

#### Procedures

Basic procedure was almost the same as Experiment 1, although all data collection was done in only one session. The flow of the experiment was following; a green square appeared on the monitor at the beginning of each trial. The square disappeared following a key-press and a sound stimulus was played back immediately. Then, a red square appeared after delivery of the sound stimulus. The participants were given 800 ms with a presentation of a red square to decide either pressing the key or not. Then, a feedback sign (either “a circle,” which means a correct sign in Japan, or “a cross” representing a false sign) was displayed on the monitor. A Key-press before presentation of the red square brought a warning sign and the response was a null and void, and then, the trial was repeated. Correction trials were introduced as the same as Experiment 1. ITI was 400 ms.

When the correct response rate reached 80% in the last 50 trials, the test period began immediately without notice and probe trials were randomly interspersed among the normal training trials at 10% probability. In this experiment, the “transfer test” was omitted. The total number of probe trial was 60 (30 probe ABB and 30 probe AAB) as the same as the birds' experiment. No feedback was given for each probe trial.

The participants were instructed that they need to make higher correct percent as much as possible to finish the experiment soon. Because they were notified that the reward was fixed (1000 JPY), depending on neither the time period nor the number of trial, we can assume that the participants tried to finish the task as soon as possible, so that a positive sign (i.e., a circle) should be a reward for them. The experimenter never told them about anything related to rules or sequences of the sound stimuli. Following the experiment, the participants answered a short questionnaire, which asked them (1) whether they noticed any rules for the sound sequences, and (2) how they discriminated the stimuli.

## Results

### Experiment 1: bengalese finches

All birds met the criterion in 33–227 sessions (103.9 ±23.2, Mean ± SE). No significant effect of sound sequence combination on session number was found; between GO-AAB / NOGO-ABB and GO-ABB / NOGO-AAB groups (98.4 ± 35.6, 110.8 ± 32.9 sessions; *w* = 7, *p* = 0.56, Wicoxon rank sum test), between MFF-MMF and FMM-FFM groups (115.0 ± 31.3, 90.0 ± 38.3 sessions; *w* = 13, *p* = 0.56, Wicoxon rank sum test). We obtained data for all birds on the transfer test; however, one bird died and another had difficulty meeting the criterion before the rule-generalization test. Therefore, only seven birds participated in the second test.

**Figure 2:**
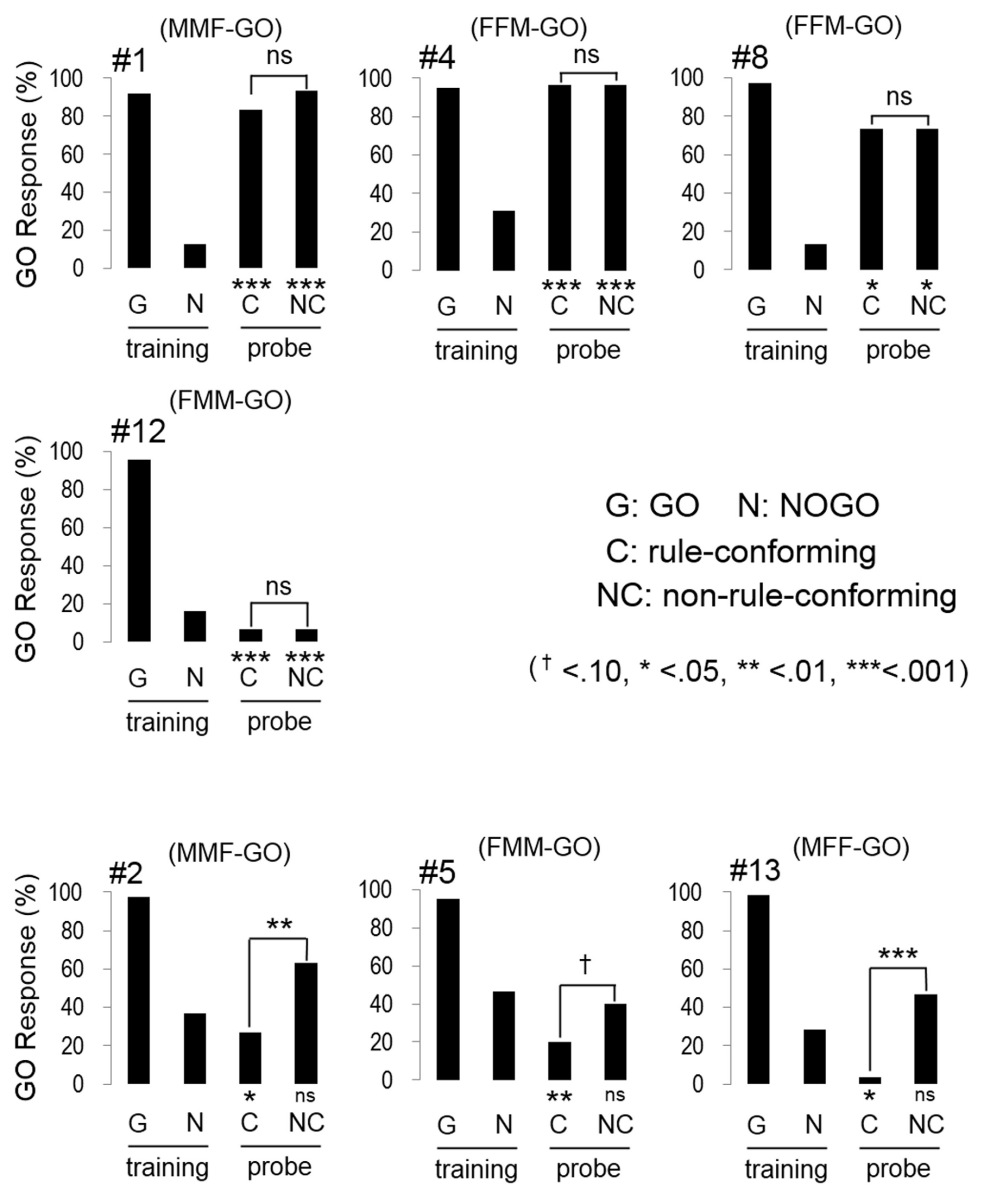
**Response of the birds in the test sessions**. Three birds (#1, #4, #8) had a strong bias to GO direction for every sound stimulus except NOGO stimulus, although #12 had a strong bias to NOGO direction for every sound stimulus except GO stimulus. The response of the remaining birds (#2, #5, #13) depended on the type of repetition sounds (e.g., FMM-GO bird showed GO response to MMF probe stimuli).

In the transfer test, the number of GO responses by all subjects was greater for the rule-conforming probe stimuli than the non-rule-conforming probe stimuli. Analysis of the pooled data revealed a significant difference in the rate of GO response between those probe types (*p* < 0.001, paired Wilcoxon test) and large *d*′ (1.31 ± 0.12, mean ± S.E.), although the value was larger for the baseline (2.55 ± 0.99). However, in the rule-generalization test, the subjects exhibited a variety of response patterns (Figure 2). As a result, the number of GO responses did not differ between rule-conforming and non-rule-conforming stimuli (mean, rule-conforming 14.6, non-rule-conforming 18.6; *p* = 0.10) and *d*′ was small (−0.39 ± 0.12) for the pooled data while the value for the baseline was 2.46 ± 0.09. The response patterns were easily categorized into 3 types. Type-1, “Go for everything except NOGO stimuli,” was observed in three birds. Responses were significantly biased toward the GO direction for both rule-conforming and non-rule-conforming probe stimuli based on a binomial test (bird #1 *p* < 0.001, *p* < 0.001; # 4 *p* < 0.001, *p* < 0.001; #8 *p* = 0.010, *p* = 0.010), and there was no difference in the GO response rate between those two probe types (#1 *p* = 0.23, #4 *p* = 1, #8 *p* = 1, chi-square test). Type-2 was “NOGO for everything except GO stimuli.” The response of bird #12 was significantly biased toward the NOGO direction (*p* < 0.001, *p* < 0.001), and there was no difference in the GO response rate between those two probe types (*p* = 1, chi-square test). Type-3 was displayed by the remaining subjects, who to some extent used “a sequence rule” as the discriminative strategy; however, it was an unexpected response. All of Type-3 birds gave more GO-Responses to *non-rule-conforming* stimuli than rule-conforming stimuli (#2 *p* = 0.003, #5 *p* = 0.09, #13 *p* = 0.008).

#### Discussion of experiment 1

The response patterns for four of seven birds (Type-1 and Type-2) on the “rule-generalization test” clearly indicated that the birds did not generalize the abstract rule regarding sequence structure (i.e., from FFM (MMF) and FMM (MFF) to AAB and ABB) in the responses to probe stimuli. Instead, they memorized a particular acoustic pattern or global sound structure (i.e., combination of 3 call sounds; like MMF or MFF) as the strategy for a GO (bird #1, #4, #8) response. Consequently, those birds produced NOGO responses to any other (including probe) stimulus (and bird #12 memorized NOGO patterns, vice versa). In contrast, the three birds that exhibited a Type-3 pattern seemed to use a “rule” in the response to probe stimuli. A possible interpretation of these results is that the birds used repeated sounds (i.e., MM or FF) as a discriminative strategy, independent of their position in the sequence (i.e., in the first half or the latter half). This interpretation seems consistent with the findings of van Heijningen et al. (2012), which suggested that zebra finches attended to the presence of repeated sounds (ten Cate and Okanoya, 2012). Another interpretation is that those birds used the “ratio of sound pattern from a particular sex included in the global sequence of the stimuli.” One more possible interpretation is the birds attended the middle sound out of the three. We do not conclude which interpretation is correct from the results.

Interestingly, the birds that adopted the simple rule (Type-3 birds) required more sessions (#2, 227 sessions; #5, 203; #13, 113) to learn the task than the birds that memorized a particular sound pattern (Type-1 and 2 birds, 33–123 sessions; *w* = 1, *p* < 0.05, Wilcoxon rank sum test). In any case, no birds used the abstract rule (i.e., AAB or ABB) as the discriminative strategy.

The next question is whether the birds' strategies are also reasonable for humans in the same operant task. The answer to this question could provide a stronger suggestion whether the birds really lacked the ability to discriminate the abstract rules or merely did not do so in the task.

### Experiment 2: humans

**Figure 3:**
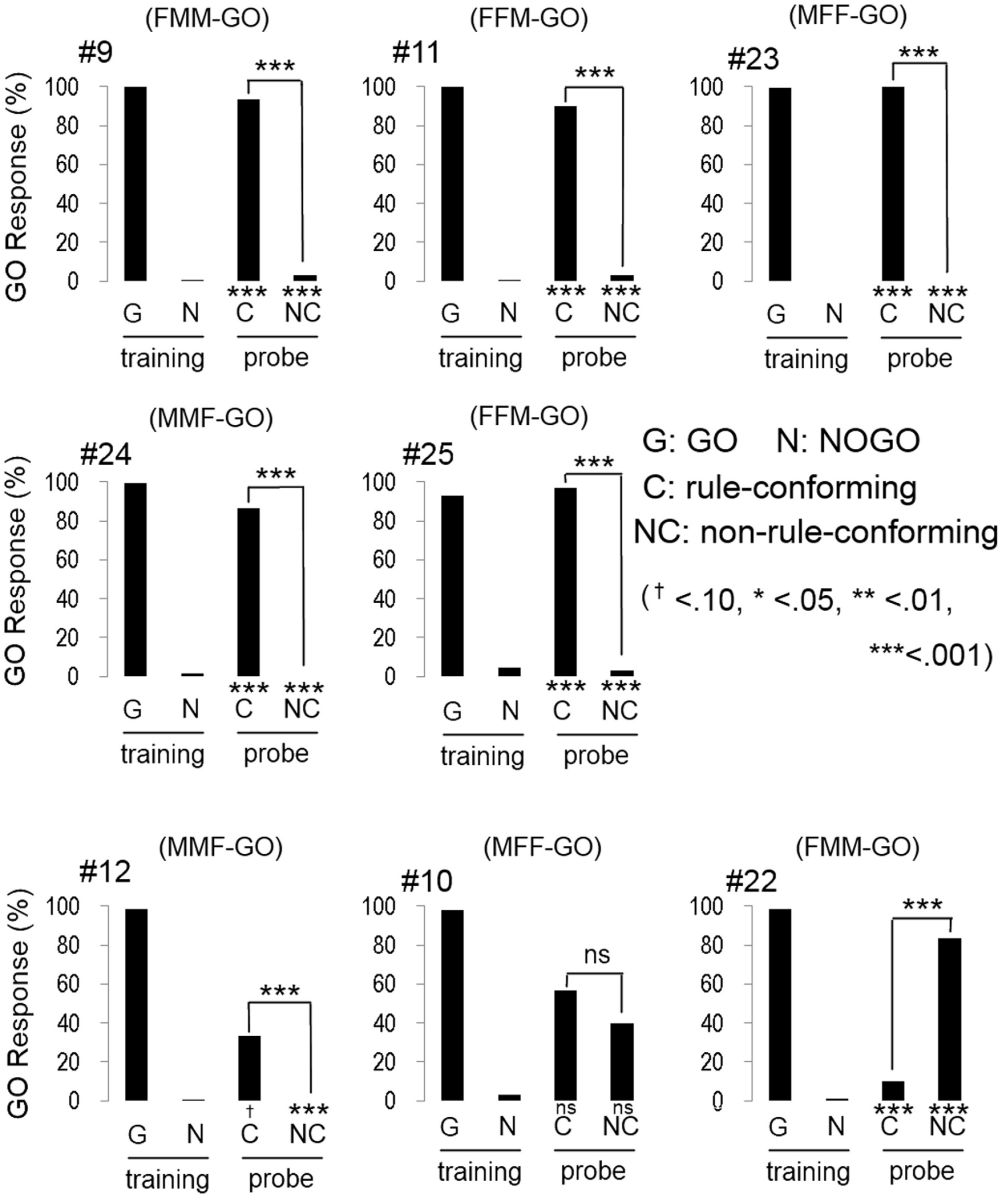
**Response of human participants in the human voice experiment**. Five participants (#9, #11, #23, #24, #25) clearly showed that they extracted the rule (AAB and ABB) from training stimuli and applied it to the response to the probe stimuli. Although the response of #12 was similar to those 5 participants, the bias to GO response for the probe stimuli was not found. Participant #10 showed unsystematic responses to the probe stimuli. The response pattern of #22 is similar to the response of birds #2, #5, and #13.

**Figure 4:**
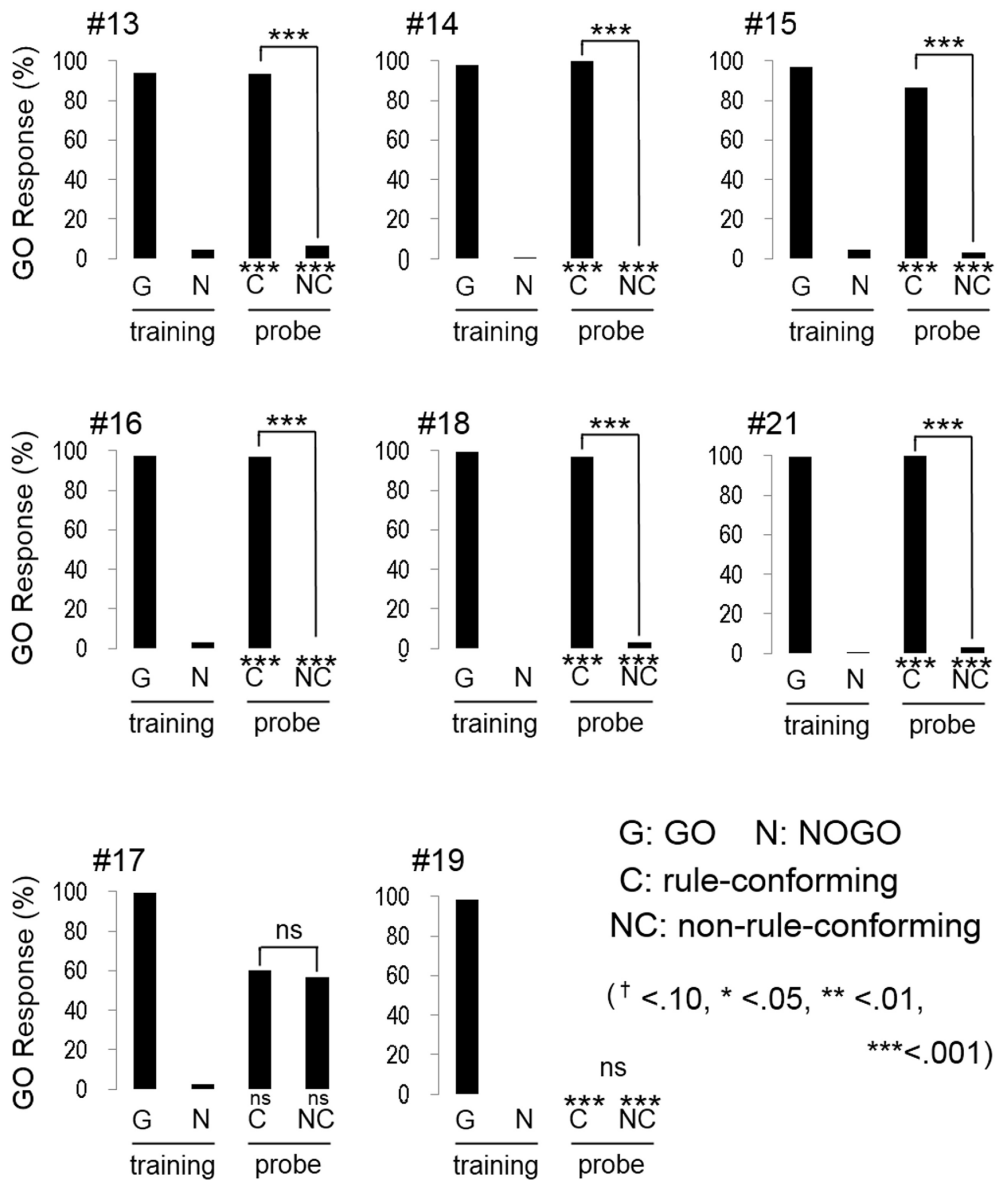
**Response of human participants in the bird vocalization experiment**. Six participants (#13, #14, #15, #16, #18, #21) extracted the abstract rule. One participant (#17) showed unsystematic response to the probe stimuli. The response pattern of #19 is quite similar to the response of bird #12 as shown in Figure 2.

All participants quickly reached the discriminative criterion (HV: 52.1 ± 0.78 trials; BV: 63.3 ± 2.57 trials; mean ± S.E.), as expected. Although the number of trials to meet the criterion in BV condition was greater than HV condition, the difference was statistically marginal (*w* = 47.5, *p* = 0.07, Wilcoxon rank sum test). Most participants (5 out of 8 in HV; Figure 3, and 6 out of 8 in BV; Figure 4) showed a strong and significant bias for the GO direction when presented with rule-conforming probe stimuli (*p* < 0.001 in each participant, binomial test) and for the NOGO direction when presented with non-rule-conforming stimuli (*p* < 0.001 in each participant); similar biases were never exhibited by the birds. The GO response rate for rule-conforming probe stimuli was significantly greater than for non-rule-conforming probe stimuli in those 11 participants (*p* < 0.001 in each participant, chi-square test). Those participants basically reported finding the “algebraic” rule (e.g., “a sound was repeated twice then a different sound follows them, or a different sound follows after repetition of a sound”) and used it as the discriminative strategy for the training and probe stimuli. However, even participants who reported not explicitly finding the rule used it as the cue for the probe stimuli. For example, participant #9 (HV: FMM-GO) reported using GO response for FMM / NOGO response for FFM (not ABB/AAB) rule as the strategy for the training stimuli; however, he also showed significantly more GO responses for MFF and NOGO responses for MMF in the probe trials (Figure 3 top left panel). Similarly, participant #13 (BV: FMM-GO) reported using the first and second sound (GO for FMX / NOGO for FFX) as the strategy; however, she showed significantly more GO responses for MFF than MMF in the probe tests (Figure 4 top left panel).

One participant (#12, HV: MMF-GO) had a greater GO response rate for the rule-conforming than the non-rule-conforming probe stimuli (*p* < 0.001, chi-square test), although he showed a bias for the NOGO direction on both rule-conforming (*p* < 0.1, binomial test) and non-rule-conforming probe stimuli (*p* < 0.001). He reported using the switch timing of sound sequence (A/B transition, not F/M) as the strategy in the training trials, but a hit-or-miss strategy for the probe stimuli. Consequently, he showed significantly different responses between rule-conforming and non-rule-conforming probe stimuli, with a NOGO bias.

Interestingly, the response patterns of two participants were similar to the birds' responses. The response pattern of participant #22, (HV: FMM-GO, Figure 3 bottom right) was similar to three birds (#2, #5, #13) and could be explained by him using repeated sounds as the discriminative strategy. It was consistent with his report that he used the number of F voice as the discriminative strategy, so that he responded more to MMF than MFF. Participant #19 (BV: FFM-GO, Figure 4 bottom middle) did not show GO response for all probe stimuli similar to bird #12. He reported categorizing the stimulus sound elements into two types and using the type of the second sound as the discriminative strategy for the training stimuli. Then, he found not only the difference between training and probe stimuli but also the consequence (i.e., no feedback) of response to the probe stimuli. Therefore, he did not pay more attention to the probe stimuli and did not show a GO response for the probe stimuli at all.

The remaining participants #10, (HV: MFF-GO) and #17 (BV: FMM-GO) responded unsystematically to the probe stimuli (no bias toward either GO nor NOGO; #10 rule-conforming *p* = 0.36, non-rule-conforming *p* = 0.58, #17, *p* = 0.58, *p* = 0.36, binomial test, and no significant difference between rule-conforming and non-rule-conforming stimuli; #10, *p* = 0.20, #17, *p* = 0.79, chi-sq. test). Although the results of those participants were similar, their discriminative strategies were different. Participant #10 reported that both the number of M and F voice and algebraic rules could be used to discriminate during the training trials, so he used both strategies for the probe trials. Participant #17 reported that probe stimuli consisted of unfamiliar sounds, so he responded to those sounds randomly.

#### Discussion of experiment 2

As expected, the results for most participants revealed that they used an abstract rule (i.e., AAB and ABB) that was acquired during the training trials for responding to probe stimuli. Although the reports of participants #9 and #13 did not indicate they explicitly found the algebraic rule, the probe tests showed they implicitly learned the rule. This finding supports previous results that humans innately extract rules from sound sequences in passive situations (Saffran et al., 1996; Marcus et al., 1999); however, the results of the probe tests were not enough to determine the discrimination ability of the participants. Interestingly, some participants reported understanding the rule (AAB and ABB) but did not use it when responding to probe stimuli.

## Discussion

The results clearly showed that the response pattern to the probe stimuli generally differed between Bengalese finches and humans. Although all patterns exhibited by the birds were also demonstrated by several human participants, in response to probe stimuli the birds never used the “global sequence rule” that was used by most human participants (Table 1).

**Table 1:** **The numbers of bird subjects and human participants that applied each strategy in the task**.

		**Using a “rule”**		**GO for everything**	**NOGO for everything**	**Unsystematic**
		**Global**	**Type of repetition**			
Birds		–	3/7	3/7	1/7	–
Humans	(HV)	6/8	1/8	–	–	1/8
	(BV)	6/8	–	–	1/8	1/8

Why did birds fail to use the rule? The most likely interpretation is that the birds could not extract the sequence rule, which seems to agree with van Heijningen et al. (2009). Comparing the study and the present one, Bengalese finches performed no better than zebra finches when engaging in the sequence rule discrimination task, although Bengalese finches produce more complex song sequences than zebra finches. In the present study, although it was not necessary to use natural sounds to allow the birds to learn such a “concept” of sequential structure, we used male (M) and female (F) calls as the sound elements, because bird calls have natural variations within those categories that could prevent the birds from using a particular sound property for discrimination. We also expected it would encourage the birds to have a flexible response for novel stimuli, and the simple stimulus sets would help the birds learn the task more quickly. However, the variation in call sounds might be insufficient for the birds to generate such flexibility; instead the birds learned two robust patterns of sound sequences [e.g., MMF/MFF not AAB/ABB; because Bengalese finches can discriminate the difference between male and female calls (Okanoya and Kimura, 1993)] and generalized it for the novel stimuli in the transfer test but they could not use it for the rule-generalizing test. If we used more complex stimuli, like song syllables or an artificial stimulus set, the results might be different. This is another possible interpretation of the present data. However, a majority of the human participants used algebraic rules in the discrimination of the probe stimuli under the same condition using M/F sound of both humans and birds. Therefore, the main and important topic of the present study is that the human strategy for discriminating ruled sound sequences is different from that of Bengalese finches even though the latter species has an ability to generate songs conforming to a kind of syntactic rule (Okanoya, 2004).

Another interpretation for why the birds did not use the abstract rule is that use of the rule might be solely more difficult than use of the other strategies, such as the Type-1, Type-2, and Type-3 in Experiment 1 for the birds (Note; even the simple rule shown in Type-3 birds required more sessions to learn than Type-1 and 2). It might be easier to memorize the acoustic pattern of the sequences than to extract rules from the sequences, especially because we used conspecific calls as the stimuli. Thus, the acoustic patterns might be easier to memorize for the birds, which may reinforce such a trend. In other words, we might be able to consider that they merely did not apply the rule on this task, but could do it when required (e.g., in more difficult task using human vocalizations, although such training could be more difficult for birds). Interestingly, human participant #19 did not apply the rule when responding to the probe stimuli although he reported finding the abstract rule on the questionnaire. These findings suggest that a bird-like response does not always mean the subject could not learn the algebraic rule from the sound sequences, although it does not allow us to consider the same thing happening in finches.

**Figure 5:**
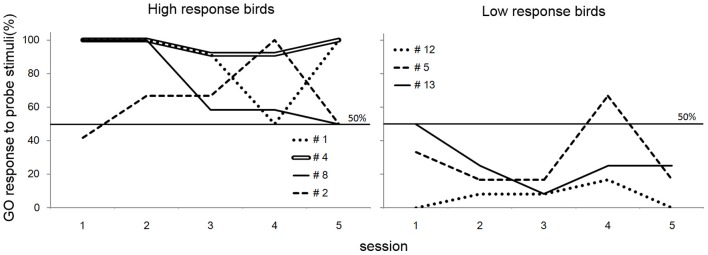
**GO response to probe stimuli of the birds in generalization test of Experiment 1**. Although fluctuations are observed, there was no obvious evidence that the birds learned the consequences of the response to the probe stimuli.

In the present experiment, we presented the bird subjects neither reinforcements nor punishments for responses to the probe stimuli. A training session, which exhibited high percentage (>85%) of correct trials, was inserted between each pair of probe sessions to prevent the birds from learning the consequences of the response to the probe stimuli. However, it might be possible that the birds learned it in the generalization test. If the birds learned the outcome of the responses to the probe stimuli, they would show a particular trend for GO response to the probe stimuli as the sessions went. However, patterns of GO response for the probe stimuli did not show a systematic change as the sessions went (Figure 5), supporting the idea that they did not explicitly learn the consequence to probe stimuli.

There is no doubt that the conditioning technique is one of the strongest tools for exploring sound sequence processing in animals, especially birds, because it is difficult to track birds' eye movements for monitoring their attention to loudspeakers as done in primate studies. For example, one study recently suggested the sound sequence generator (i.e., the song nervous system) might be related to learning and production of ruled sequences using an operant method (Yamazaki et al., 2012). However, the methods of the present experiment were not enough to show a critical threshold of ability for sound sequence processing, because the response to the probe stimuli merely showed the strategy of each subject but did not always reflect the competence of rule extraction in human subjects (and maybe birds). In contrast, some studies (Stripling et al., 2003; Abe and Watanabe, 2011) have used spontaneous activities (vocalization, hopping, etc.) to determine whether the subjects detected differences between sound stimuli. This approach would be better if it were applicable for any stimulus and environment (however, See, Beckers et al., 2012).

Another approach to this issue might be electrophysiological recordings of neural activity as has been examined in humans (e.g., Sun et al., 2012). Beckers and Gahr (2012) found that neurons from a higher-order auditory area in zebra finches showed “mismatch negativity (e.g., Näätänen, 2008)” like responses to irregular sound sequences. In addition, researchers have developed a habituation-dishabituation method to examine the auditory memory of birds using the multi-unit activity of neurons (Chew et al., 1995), suggesting that such methods may be useful for testing sound sequence processing in birds.

In conclusion, our findings suggest that the cognitive process of rule extraction from sound sequences differs between songbirds and humans, although testing with the present operant method did not provide critical evidence that the birds do not have the ability demonstrated by the humans. Although songbirds are unique species as experimental and operational animal models of the evolution of language, we believe the data show that humans have a stronger tendency to extract sequential rules; thus, this skill should be easier to perform for the cognitive system of humans than that of songbirds. The nature of this disposition is one of the peculiarities that enable humans to manipulate language.

### Conflict of interest statement

The authors declare that the research was conducted in the absence of any commercial or financial relationships that could be construed as a potential conflict of interest.
